# Identification of the original species of cubilose based on DNA barcode

**DOI:** 10.1080/23802359.2019.1666681

**Published:** 2019-09-19

**Authors:** Shasha Wang, Jinlin Guo, Feixia Hou

**Affiliations:** Pharmacy College, Chengdu University of Traditional Chinese Medicine, Chengdu, Sichuan, China

**Keywords:** Cubilose, original species, DNA barcoding, cytb

## Abstract

Cubilose, a valuable traditional Chinese medicine, is mainly composed of the saliva by several species of *Aerodramus* or *Collocalia* in the Apodidae. Due to rarity, high economic value and huge market demand, its fake or adulteration is frequently found in the market. Therefore, it is urgent to establish a simple and accurate method for authenticating cubilose. DNA barcoding, which is an easy, quick and reliable method, is widely used to trace the origin of traditional Chinese medicine. For identifying the original species of cubilose, cytb gene of 18 cubilose samples including 15 officer cubilose and 3 feather cubilose were amplified and entered into the GenBank database using the BLAST search tool. The genetic distances among 18 cubilose samples were calculated based on the Kimura two parameter (K2P) model. To construct the reference database, 18 cytb sequences of *Aerodramus* or *Collocalia* were downloaded from GenBank. The neighbor-joining (NJ) and unweighted pair group method with arithmetic average (UPGMA) trees were constructed based on sequences from GenBank and our dataset. Blast analysis showed that all cubilose samples had the highest similarity with *A. fuciphagus*, and the sequence similarity reached over 99%. Genetic distance of 18 cubilose samples ranged from 0.000–0.010. Trees constructed by NJ and UPGMA gave similar topology: all cubilose samples clustered together with *A. fuciphagus.* These result demonstrated that the original species of all 18 cubilose samples were *A. fuciphagus,* and cytb gene is a good candidate for identifying cubilose.

Cubilose is from the nest of several species of *Aerodramus* or *Collocalia* in the Apodidae, which is made by their saliva secretion (Green 1885). It is predominately produced in Southeast Asia including Indonesia, Malaysia, Thailand and Vietnam (Chan et al. 2013). Cubilose has been served as functional food in Asia for over 1000 years, and has tremendous market value. Cubilose contains a lot of proteins and bioactive substances (Yang et al. 2014), and is a high health care product as famous as ginseng and deer antler. According to traditional application, the intake of cubilose could strengthen digestive system, repair lung function, improve immune system and enhance skin repairing (Zhao 1765). Commercial cubilose mainly include officer cubilose, feather cubilose and grass cubilose. Officer cubilose contain almost no feathers, while feather cubilose has a lot of feathers. Grass cubilose is mainly composed of plants. Officer cubilose could be divided into house cubilose and cave cubilose by nesting site, and white cubilose, yellow cubilose and red cubilose by color. It was reported that officer cubilose, feather cubilose and grass cubilose were produced by different swiftlets (Wang et al. 2015). Due to its rarity, high nutritive value and huge market demand, the price of cubilose is very expensive. Rich profit and lack of scientific identification criteria lead to the quality of cubilose vary tremendously. For standardizing the market and reasonably exploiting resource, it is urgent to establish an easy and reliable method for authenticating cubilose.

Because of different swiftlets producing different cubilose, the price and quality of cubilose are impacted not only by color, ingredient and shape, but also by its original species (Koon and Cranbrook 2003). The quality of cubilose is traditionally regarded as closely related to locality, for example, cubilose from Hoi An of Vietnam is superior to Indonesia and Thailand, which may be correlated with different swiftlets (Chen et al. 2017). The ornithologist classified the nest-bearing swiftlets into two main groups: *Aerodramus* including *Aerodramus fuciphagus*, *Aerodramus maximus*, *Aerodramus germani*, *Aerodramus sunicolor* and *Aerodramus francicus*, and *Collocalia* including *Collocalia troglodytes*, *Collocalia linchi* and *Collocalia esculenta* (Jiang 2016). It was reported that *A. fuciphagus*, *A. maximus* and *Collocalia* genus produced officer cubilose, feather cubilose and grass cubilose, respectively (Lin 2010). At present, the study about cubilose mainly involves pharmacological effect (Wong et al. 2018; Mei et al. 2018; Ruan et al. 2019), variety investigation (Lai et al. 2005), quality research (Wang et al. 2013; Shangguan et al. 2018) and crude drug identification (Hou 2010; Guo 2014; Kong et al. 2015; Yu 2015). The currently main authentication methods for cubilose, such as protein electrophoresis (Hou 2010), infrared spectrometry (Guo 2014), H-nuclear magnetic resonance (Kong et al. 2015), liquid chromatography quadrupole time of flight tandem mass (LC/Q/TOF) and Raman spectroscopy (Yu 2015), which could indicate the authenticity and purity of cubilose by detecting the differences in chemical components between cubilose and its counterfeits, cannot identify its genetic source. Therefore, identification of the genetic source of cubilose would be more conducive to ensure its quality. Nicotinamide adenine dinucleotide dehydrogenase subunit 2 (ND2) and mitochondrial cytochrome oxidase I (COI) have been used to identify of the original species of cubilose (Wang et al. 2015; Diao et al. 2017). Mitochondrial cytochrome b (cytb) gene, which is characterized by conservative composition, matrilineal inheritance and no recombination, is used as DNA barcode to identify species (Koon and Cranbrook 2003). Cytb was also widely used to analyze interspecies genetic difference of bird and the evolutionary classification of *Aerodramus* (Lee et al. 1996; Thomassen et al. 2003; Thomassen et al. 2005). In order to explore genetic differences of cubilose from producing areas and different kinds, search for more efficient DNA identification fragment, and provide the theoretical basis for the traceability and quality evaluation, cytb gene was applied to identify the origin of cubilose.

A total of 18 samples including 15 officer cubilose and 3 feather cubilose were randomly purchased from 14 shops in Chengdu city lotus pond TCM market. The sample information was showed in Table 1. All corresponding voucher samples were deposited in the herbarium of Chengdu University of Traditional Chinese Medicine. Genomic DNA was extracted using oral swab genomic DNA extraction kit. Cytb sequences of 18 cubilose samples were amplifided using the forward primer ND5 (5′-TAGCTAGGATCTTTCGCCCT-3′) (Koon and Cranbrook 2003) and reverse primer H15709 (5′-GGCATATGCGAATARGAARTATCA-3′) (Lin 2010). PCR amplification was performed in a total volume 25 μL containing 12.5 μL 2 × Taq master mix buffer, 15 ng genomic DNA and 0.1 μ&Mu; of each primer. PCR amplification program was 94 °C for 2 min, followed by 35 cycles of 94 °C 30 s, 55 °C 30 s, 72 °C 1 min, with a final elongation of 72 °C for 10 min. All PCR products were checked by electrophoresis in a 1.5% agarose gel, then purified and bi-directional sequenced at Invitrogen^TM^ Life Technology, Shanghai, China. The indices for the evaluation of a DNA-barcoding include successful PCR amplification and sequencing (Yan et al. 2013). Although cubilose samples in this study had undergone manufacturing processes and were stored at room temperature, the result indicated that genomic DNA extracted from commodity cubilose were qualified and sufficient for cytb sequences amplification. The success rate in PCR amplification and sequencing was 100%. Cytb sequences were checked and merged using the CondonCode Aligner V 3.61 (CodonCode Co., USA). Eighteen sequences were aligned with a consensus length of 792 bp, and all sequences were submitted to GenBank with accession numbers MN124134-151 (Table 1). There were no insertions, deletions or stop codons within the analyzed sequences. DnaSP V.5.10.01 (Librado and Rozas 2009) analysis showed that there were 13 variable sites including 4 singleton variable sites and 9 parsimony informative sites. On average, the nucleotide composition of all the sequences was A = 28.1%, T = 22.2%, G = 13.1%, C = 36.6%.

**Table 1.** Sample information of cubilose used in this study and blast search result.

Sample ID	GenBank No.	Variety	Color	Nesting site	Producing area	Original species	GenBank No. of origin species	Similarity
**OC1**	MN124134	Officer cubilose	White	house	Malaysia	*A. fuciphagus*	KX944190, KR818754, KR818758	99.75%
**OC2**	MN124135						KR818759, KR818756	100%
**OC3**	MN124136							99.87%
**OC4**	MN124137						KX944190, KR818754, KR818758	99.75%
**OC5**	MN124138						KR818754, KR818758	100%
**OC6**	MN124139						KX944195-96, KX944184-86, 88, KR818757, AY135631-32	
**OC7**	MN124140							
**OC8**	MN124141						KR818754, KR818758	
**OC9**	MN124142				Indonesia		KR818755, KX944187	
**OC10**	MN124143		Yellow				KX944195-96, KX944184-86, 88, KR818757, AY135631-32	
**OC11**	MN124144						KX944190, KR818754, KR818758	99.87%
**OC12**	MN124145						KR818755, KX944187	100%
**OC13**	MN124146		Red					
**OC14**	MN124147		Yellow	cave	Thailand		KX944189, KX944190	99.49%
**OC15**	MN124148				Malaysia		KX944189	99.87%
**FC1**	MN124149	Feather cubilose					KX944190, KR818754, KR818758	99.75%
**FC2**	MN124150						KX944195-96, KX944184-86, 88, KR818757, AY135631-32	100%
**FC3**	MN124151				Indonesia			

Eighteen cytb sequences were entered into the GenBank database using the BLAST program V 2.2.17 (Ross et al. 2008). The results showed that all sequences of cubilose samples had the highest similarity with *A. fuciphagus*. The sequence similarity reached over 99% (Table 1). Genetic distance of 18 cubilose samples ranged from 0.000-0.010, indicating that all cubilose were from the same species. The results of blast and genetic distance analysis suggested that the original species of all 18 cubilose samples were *A. fuciphagus.* To construct the reference database, 18 cytb sequences of *Aerodramus* or *Collocalia* were downloaded from GenBank (accession numbers were shown in Figure 1). The neighbor-joining (NJ) and unweighted pair group method with arithmetic average (UPGMA) trees were constructed with 1000 bootstrap replicated (Felsenstein 1985) based on sequences from GenBank and our dataset. NJ and UPGMA phylogenetic trees showed a similar topology: all cubilose samples clustered together with *A. fuciphagus.* The result further demonstrated that the original species of all 18 cubilose samples were *A. fuciphagus*, and cytb gene was a good candidate for identifying cubilose.

**Figure 1.**
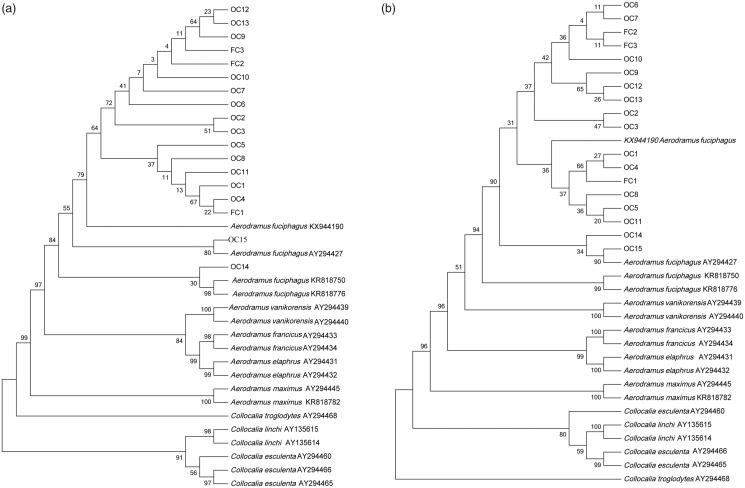
Phylogenetic trees based on cytb sequences with 1000 bootstrap replicates. (a) NJ tree; (b) UPGMA.

DNA barcoding, a diagnostic technique for species identification using a short and standardized DNA, is the hot field and prosperous direction of biological classification and identification. The main purpose of DNA barcoding research is to identify species and discover new species (Herbert et al. 2004a, 2004b), and it has great advantages in identifying samples with unknown source. In recent years, DNA barcoding technique has been successfully applied in species tracing of processed meat and seafood (Willette et al. 2017; Quinto et al. 2016), which provide guarantee for food quality supervision. From picking to processing products, cubilose go through complicated process including removing feather and shaping. Finished products with very similar shape cannot be identified through morphological characteristics. This study identified 18 cubilose samples including three producing areas, five color, two nesting sites based on DNA barcoding. Blast, genetic distance and phylogenetic tree analysis indicated that the original species of all cubilose samples were *A. fuciphagus*. This result was consistent with that of previous studies (Lin 2010; Diao et al. 2017), which verified the correctness of the identification result and the feasibility of using DNA barcoding technique to identify cubilose and its original species. Compared with previous literatures, cytb gene is more suitable for rapid identification of origin and authenticity of cubilose because of the advantages of moderate conservatism and easily acquired.

This study indicated that the original species of cubilose from different producing areas (country), color (white, yellow, red) and nesting sites (house, cave) had no regularity. That is, cytb gene of cubilose from different producing areas, color and nesting sites has no interspecific difference, so do COI (Diao et al. 2017) and ND2 gene (Wang et al. 2015). Cubilose is found in Indonesia, Malaysia, Thailand, Vietnam, Philippines and China, with Indonesia accounting for 80% of global production, Malaysia 13% and Thailand about 5%. Only 16 companies in Malaysia and 6 in Indonesia were allowed to export cubilose into China (Diao et al. 2017). Cubilose entering China through illegal channels and passengers carrying has great food safety and biosafety risks. In order to protect the rights of consumers, solve the problem that mark producing area does not match the actual producing area on the market, the sample size of cubilose needs to be expanded, and genetic differences of cubilose with different producing areas and identical origin species need to be explored.

In conclusion, this study developed a DNA barcoding technique to identify the original species of cubilose, and provided a theoretical reference for evaluating cubilose quality. It also provides theoretical basis for exploiting cubilose resources reasonably and standardize cubilose market.
